# Increased Risk of Temporomandibular Joint Disorder in Patients with Rheumatoid Arthritis: A Longitudinal Follow-Up Study

**DOI:** 10.3390/jcm9093005

**Published:** 2020-09-17

**Authors:** Soo-Hwan Byun, Chanyang Min, Hyo-Geun Choi, Seok-Jin Hong

**Affiliations:** 1 Department of Oral & Maxillofacial Surgery, Dentistry, Hallym University College of Medicine, Anyang 14068, Korea; purheit@daum.net; 2 Research Center of Clinical Dentistry, Hallym University Clinical Dentistry Graduate School, Chuncheon 24252, Korea; 3 Hallym Data Science Laboratory, Hallym University College of Medicine, Anyang 14068, Korea; joicemin@naver.com; 4 Department of Otorhinolaryngology-Head & Neck Surgery, Hallym University College of Medicine, Anyang 14068, Korea; 5 Department of Otorhinolaryngology-Head & Neck Surgery, Hallym University College of Medicine, Dongtan 18450, Korea

**Keywords:** temporomandibular joint, TMJ, TMD, disorder, rheumatoid arthritis

## Abstract

We evaluated the incidence of temporomandibular disorder (TMD) in patients with rheumatoid arthritis (RA) and examined the association between TMD and RA, through longitudinal follow-up. Population data from the Korean National Health Insurance Service-Health Screening Cohort from 2002 to 2015 was used. From 514,866 subjects, 3122 with RA were matched with 12,488 controls in a 1:4 ratio. The crude and adjusted models (for obesity, smoking, alcohol consumption, blood pressure, blood glucose, total cholesterol, and Charlson Comorbidity Index scores) were calculated. Chi-square tests, Kaplan-Meier (KM) analysis, and two-tailed analyses were used for statistical analysis. Stratified Cox proportional hazard models were used to assess the hazard ratios (HR) and 95% confidence intervals (CI) for TMD in the RA group, compared to those in the control group. The adjusted HR for TMD in RA was 2.52 (95% CI = 1.70–3.74), compared to the control group. The results were consistent with the subgroup analyses, according to age and sex, except in men older than 60 years of age. KM analysis showed similar results. Hence, we found that patients with RA have a higher risk of TMD, and should be observed for symptoms of the initial stage of TMD to prevent the risk of aggravation.

## 1. Introduction

Rheumatoid arthritis (RA) is an autoimmune disease that occurs not only in the elderly, but also in younger patients [1]. RA is one of the most prevalent chronic inflammatory diseases [2]. It is a destructive, progressive, and disabling condition affecting over 1% of the world’s population [3]. Females are affected approximately three times more than the males, with a peak prevalence between 35 and 50 years of age [4,5]. Smoking is the one of the environmental risk factors that doubles the risk of RA [6,7]. Other environmental risk factors include coffee consumption, vitamin D status, alcohol consumption, and poor socio-economic status, although there is no strong supporting evidence with respect to the influence of these factors [8,9]. There is also inadequate evidence regarding the role of dietary control in preventing RA [10]. The clinical diagnosis of RA is based on the symptoms of chronic inflammatory arthritis [11]. Laboratory investigations for RA include either erythrocyte sedimentation rate and C-reactive protein levels or both [12]. Previous studies reported that degeneration of the joint in patients with RA, develops within 3 years after the onset of the condition, and aggravates progressively [13,14]. Hence, early detection of RA is essential, since early active medical treatment could significantly influence the prognosis [15,16]. Extensive changes seen on radiographs reveal that RA is not easily controlled, and aggressive progression of joint degeneration requires comprehensive treatment [12]. Tumor necrosis factor inhibitors and other agents heralded a so-called therapeutic revolution, improving the prognosis in patients with RA [12]. However, improved disease outcomes preceded the development of these biological agents, reflecting the efficacy of early use of conventional drugs, ambitious treatment goals, and better management of comorbidities. About 5 to 15% of patients with RA achieved drug-free remission in historical cohorts [17,18]. Recent treatment methods aim to increase the frequency of drug-free remission and achieve long-term disease modification [2]. Clinical symptoms of temporomandibular disorder (TMD) are seen in 2 to 16% of the population [19,20]. Anatomical involvement of the temporomandibular joint (TMJ) is observed in 35 to 94% of patients with TMD [21,22]. Pain occurs in 10 to 25% of those affected; however, fewer than 7% of patients with TMD require treatment [23]. The Research Diagnostic Criteria for TMD (RDC/TMD) were introduced on the basis of the essential principles of a dual-axis system reflecting the biopsychosocial model, a clear operationalization for better reliability, and allows multiple diagnoses [24]. The criteria were demonstrated in the various validation research on RDC/TMD. The most recent diagnostic criteria (Diagnostic Criteria for TMD, DC/TMD) upgraded the principles and diagnostic protocols for evaluating the psychosocial aspect [25]. The pathophysiology of TMD is complex and multifactorial. The pain-sensitive structures of TMJ include the discal ligament, capsule, and posterior disc attachment, which are highly vascular and innervated. Fibrocartilage shows a better ability for repair, compared to hyaline cartilage. However, when the TMJ is unable to repair itself, joint degeneration develops [19,26]. This could be considered an inflammatory or degenerative disorder, internal joint derangement, and muscular disorder. Greene et al. suggested that TMD-related symptoms could be improved with conservative and nonsurgical treatments [27]. They suggested that if painful TMD could be treated successfully by conservative and nonsurgical methods, surgical correction of the internal derangement should be avoided [27].

RA usually affects the large joints of the hands and legs. It can also affect the TMJ. However, TMJ involvement in RA is not often evaluated, because involvement of the other joints precedes that of the TMJ [28,29]. Osseous changes due to RA at the TMJ were observed on radiographs [30,31,32]. Goupille et al. reported that erosion of the condylar area and glenoid fossa, decreased joint space, and flattening of the articular eminence were found in patients with RA [31]. Although the previous studies did not analyze the incidence of TMD in patients with RA, they reported a positive relationship between TMD and RA in small population cohorts. The purpose of this study was to evaluate the incidence of TMD in patients with RA and identify the association between TMD and RA through a longitudinal follow-up study, using the population data from a national health screening cohort.

## 2. Materials and Methods

### 2.1. Study Population

This study was approved by the ethics committee of Hallym University (2019-10-023), and the need for written informed consent was waived by the Institutional Review Board. All analyses adhered to the guidelines and regulations of the Ethics Committee of Hallym University. A detailed description of The Korean National Health Insurance Service–Health Screening Cohort data is described elsewhere [33,34]. 

### 2.2. Definition of Rheumatoid Arthritis 

RA was defined on the basis of previous studies that reported the prevalence and incidence of RA in Korea [35,36]. RA was diagnosed on the basis of ICD-10 codes (M05 or M06) and the identification of a prescription for a biological agent or any disease-modifying, anti-rheumatic drug. 

### 2.3. Definition of Temporomandibular Joint Disorder 

TMD was defined in patients who were diagnosed with ICD-10 code K07.6. For the accuracy of diagnosis, we only selected participants who were treated ≥2 times, with the diagnosis of TMD.

### 2.4. Participant Selection

**Figure 1:**
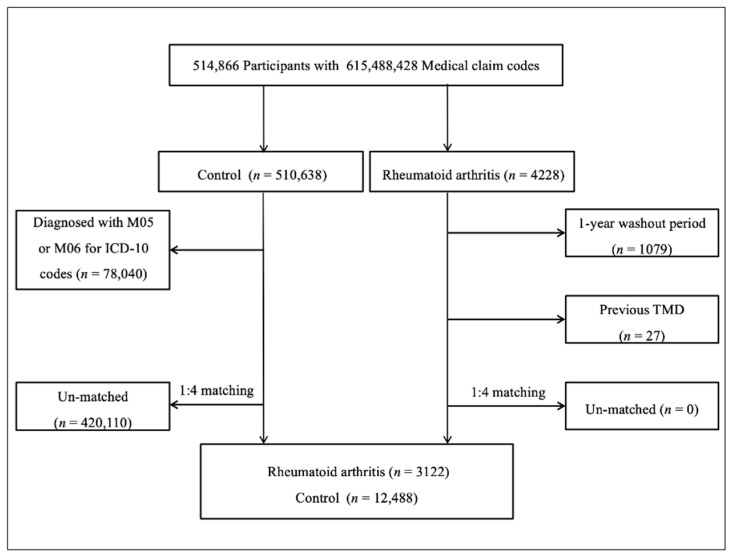
Schematic flowchart of the participant selection process used in the present study.

Patients with RA (*n* = 4228) were selected from 514,866 participants with 615488428 medical claim codes from 2002 through 2015. The control group comprised participants not diagnosed with RA (*n* = 510,638). Patients with RA diagnosed in 2002 were excluded to ensure selection of patients diagnosed for the first time (washout period, *n* = 1079). Participants in the control group were excluded if they were diagnosed with either an M05 or M06 ICD-10 code (*n* = 78,040). Patients with RA were matched with controls for age, sex, income, and region of residence, in a ratio of 1:4 [37]. To prevent selection bias during the matching process, the controls were sorted using a random number order and were then selected from top to bottom [38]. It was assumed that the matched controls were evaluated simultaneously with each matched participant with RA (index date). In both the RA and control groups, participants who had a history of TMD before the index date were also excluded. In the RA group, 27 participants were excluded. During the matching process, 420,110 of the controls were excluded. Finally, 3122 patients with RA were matched with 12,488 controls for age, sex, income, and region of residence in a ratio of 1:4 (Figure 1).

### 2.5. Covariates

Participants were divided into a total of 10 age groups of 5-year intervals: 40–44, 45–49, 50–54, 55–59, 60–64, 65–69, 70–74, 75–79, 80–84, and ≥85 years old. Income groups were categorized into 5 classes, with class 1 indicating the lowest income and class 5 indicating the highest income. Region of residence was categorized into urban (Seoul, Busan, Daegu, Incheon, Gwangju, Daejeon, and Ulsan) and rural (Gyeonggi, Gangwon, Chungcheongbuk, Chungcheongnam, Jeollabuk, Jeollanam, Gyeongsangbuk, Gyeongsangnam, and Jeju) areas.

Tobacco smoking was categorized on the basis of the participants’ current smoking status (nonsmoker, past smoker, and current smoker) [39,40]. Alcohol consumption was categorized on the basis of the frequency of alcohol consumption (<1 time a week and ≥1 time a week). Obesity was measured using body mass index (BMI, kg/m^2^). BMI was categorized on the basis of the Asia-Pacific criteria, following the Western Pacific Regional Office (WPRO) 2000 as <18.5 kg/m^2^ (underweight), ≥18.5 to <23 kg/m^2^ (normal), ≥23 to <25 kg/m^2^ (overweight), ≥25 to <30 kg/m^2^ (obese I), and ≥30 kg/m^2^ (obese II) [41]. Systolic blood pressure, diastolic blood pressure, fasting blood glucose, and total cholesterol were also measured.

The Charlson Comorbidity Index (CCI) is widely used to measure the disease burden using 17 comorbidities. A score was assigned to each participant, depending on the severity and number of diseases. The CCI was measured as a continuous variable (0 [no comorbidities] through 29 [multiple comorbidities]) [42,43].

### 2.6. Statistical Analyses

Chi-square tests were used to compare the general characteristics between the RA and the control groups. Stratified Cox proportional hazard models were used to assess the hazard ratios (HR) and 95% CI for TMD in the RA group, compared with those in the control group. In this analysis, crude (simple) and adjusted (for obesity, smoking, alcohol consumption, systolic blood pressure, diastolic blood pressure, fasting blood glucose, total cholesterol, and CCI score) models were used. Age, sex, income, and region of residence were stratified. Kaplan-Meier (KM) analysis and the log-rank test were used to analyze the cumulative probability of TJD in the RA group, compared to that in the control group. For the subgroup analyses, we divided the participants by age and sex (<60-years old and ≥60-years old; men and women) and analyzed the crude and adjusted models. Two-tailed analysis was performed in the Cox proportional hazard model, and significance was defined as a *p*-value < 0.05. SAS version 9.4 (SAS Institute Inc., Cary, NC, USA) was used for the statistical analyses.

## 3. Results

The general characteristics with respect to age, sex, income, and region of residence were similar between the RA and control groups, due to cross-matching (Table 1), while the other general characteristics like obesity, smoking, drinking alcohol, blood pressure, fasting blood glucose, and total cholesterol varied between groups.

**Figure 2:**
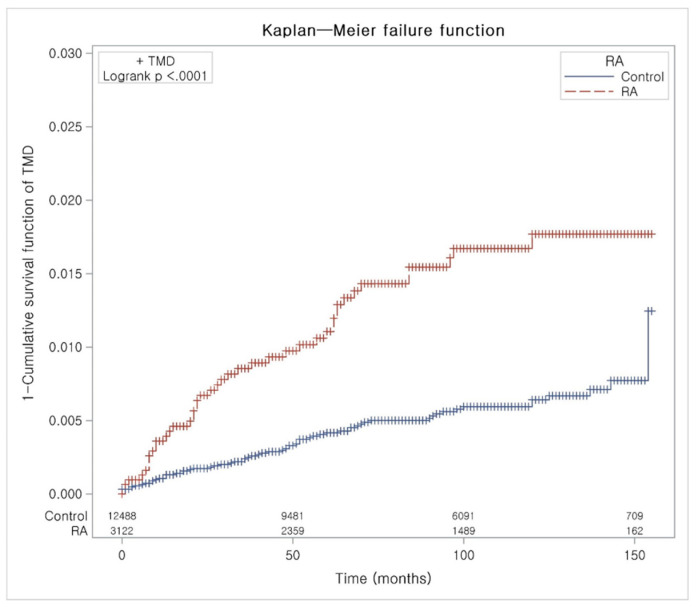
Kaplan-Meier curve of temporomandibular disorder in patients with rheumatoid arthritis. This is a 1 – survival function curve. TMD—temporomandibular disorder; and RA—Rheumatoid arthritis.

The adjusted HR for TMD in the RA group was 2.52 (95% CI = 1.70–3.74), compared to the control group (Table 2). The results were consistent in the subgroup analyses, according to age and sex, except in men <60 years old. The KM analysis showed similar results (Figure 2).

## 4. Discussion

This study revealed that the adjusted HR for TMD in patients with RA was significantly higher than in the control group. These findings were clinically significant because the degenerative changes due to RA could induce pain and functional incompetence, which affected the range of mouth opening, mastication, and speech. The TMJ affected by RA might encompass joint stiffness, pain, swelling, changes in the jaw alignment, and difficulty in opening the mouth [44]. TMJ pain could have a negative effect on the normal activities and quality of life in patients with RA [45,46]. These findings were similar to those of previous studies, in which degenerative bone changes were observed on radiographic or magnetic resonance imaging, in patients with TMJ and RA (MRI) [47,48,49]. Meanwhile, Uchiyama et al. demonstrated that the incidence of bony deformation in the mandibular condyle was not related to the duration of RA or changes in the other joints [50]. However, this study did not focus on TMD, but on bony deformation in the condylar area. Even in the absence of bony deformation visible on MRI, TMD symptoms might occur due to inflammation or myofascial pain. The results in our study were consistent in the subgroup analyses, except in the group of men <60 years old. This inconsistent result might be explained by the small number of subjects in the male <60 years old group. 

The association between RA and TMD might be explained by three pathologies. First, in patients with RA, the articular surface of the condyle was covered by inflammatory granulation, which caused destruction of the osseous structures in the joint area [51]. The pathological change began with deterioration of the articular cartilage, due to an excessive load above the functional adaptive remodeling, with concomitant activation of the degraded proteoglycans and proteolytic enzymes in the synovial fluid [52]. This could lead to a secondary inflammatory response with degradation of the joint components, including TMJ [19,53]. The inflammatory response might also influence the manifestation of muscle disorders, such as myofascial pain. A previous study demonstrated that inflammation occurred in the RA affected joints, prior to the osseous changes. Even in the initial stage of RA, localized osteoarthritis might occur as an inflammatory disease in the TMJ area [52]. This initial inflammation might induce mild swelling and limit movement within the TMJ area, in patients with RA. Nevertheless, the diagnosis of degenerative changes in the TMJ area is usually detected at a late stage, through clinical examination. 

Second, the symptoms of patients with RA are usually controlled by immunosuppressive drugs and non-steroidal anti-inflammatory drugs (NSAIDs). The use of these drugs and the increased bone resorption due to RA could induce a significant deterioration of the anatomic structures, such as the TMJ [3]. Third, even if the TMJ was not directly influenced by RA, masticatory muscles might be affected by the clenching and bruxism, due to the psychological aspect of RA, which is a chronic disease [54].

The aim of treatment in RA is to decrease inflammation in the joints, reduce pain, and prevent joint damage. Treatment of RA, such as NSAID therapy and arthrocentesis in the TMJ area is similar to that in other joints. NSAID medications were used during the acute pain period, until the pain subsided. Trieger et al. reported the effect of arthrocentesis in the treatment of TMD in patients with RA, and demonstrated that this method was useful for the short-term management of TMD symptoms [55]. An occlusal stabilization splint might also have a positive effect on the TMD [56]. 

This study had some strengths. First, we used a large population-based data set, the Korean National Health Insurance Service–Health Screening Cohort, which is representative of the Korean population. There are only a few studies regarding the association between TMD and RA. However, most of them were based on data from small populations. Moreover, in our study, the participants with TMD were followed for a maximum of 13 years. Second, the diagnosis of TMD in Korea is commonly based on the International Classification of Diseases (ICD)-10, which has a reliable criterion. Third, the data were collated by experienced clinicians. Data of the previous studies were mostly collected by trained researchers using questionnaires, rather than by doctors. Lastly, multiple confounding factors were adjusted to reduce the surveillance bias. This study included various influential factors, such as socio-economic status, obesity, smoking, alcohol consumption, blood pressure, glucose level, and cholesterol level. 

However, this study also had a few limitations. First, there were a small number of participants in the subgroups, after cross-matching. Although this study was initiated with 514,866 participants, there were only 2045 male participants aged <60 years old, and 2160 male participants aged ≥60 years old. These small numbers in participants might led to less reliable results in the subgroup analysis. Second, this study endeavored to collect and adjust as many confounding factors as possible. However, it was impossible to adjust for all the factors, since all factors were not included in the dataset.

## 5. Conclusions

RA patients might have pain/swelling and an inadequate range of motion in their joints. These could adversely affect normal activities. RA is commonly observed in other joints before the TMJ is involved. If the symptoms of TMD and RA occur simultaneously, it could lead to severe difficulty for the patient. Due to the important role of the TMJ in mastication, swallowing, and phonation, we recommend that patients with RA should be observed for TMD symptoms at the initial stage to prevent the risk of aggravation. Appropriate diagnostic criteria should be applied for the accurate diagnosis of TMD and RA.

## Figures and Tables

**Table 1:** General characteristics of the participants.

Characteristics		Total Participants		
		Rheumatoid Arthritis (*n*, %)	Control (*n*, %)	*p*-Value
Age (years old)				1.000
	40–44	109 (3.5)	436 (3.5)	
	45–49	364 (11.7)	1456 (11.7)	
	50–54	679 (21.8)	2716 (21.8)	
	55–59	585 (18.7)	2340 (18.7)	
	60–64	546 (17.5)	2184 (17.5)	
	65–69	427 (13.7)	1708 (13.7)	
	70–74	240 (7.7)	960 (7.7)	
	75–79	132 (4.2)	528 (4.2)	
	80–84	35 (1.1)	140 (1.1)	
	85+	5 (0.2)	20 (0.2)	
Sex				1.000
	Male	841 (26.9)	3364 (26.9)	
	Female	2281 (73.1)	9124 (73.1)	
Income				1.000
	1 (lowest)	526 (16.9)	2104 (16.9)	
	2	472 (15.1)	1888 (15.1)	
	3	536 (17.2)	2144 (17.2)	
	4	661 (21.2)	2644 (21.2)	
	5 (highest)	927 (29.7)	3708 (29.7)	
Region of residence				1.000
	Urban	1344 (43.1)	5376 (43.1)	
	Rural	1778 (57.0)	7112 (57.0)	
Obesity †				
	Underweight	61 (2.0)	282 (2.3)	0.334
	Normal	1189 (38.1)	4559 (36.5)	
	Overweight	821 (26.3)	3368 (27.0)	
	Obese I	960 (30.8)	3862 (30.9)	
	Obese II	91 (2.9)	417 (3.3)	
Smoking status				0.570
	Nonsmoker	2562 (82.1)	10,348 (82.9)	
	Past smoker	213 (6.8)	817 (6.5)	
	Current smoker	347 (11.1)	1323 (10.6)	
Alcohol consumption				0.081
	<1 time a week	2516 (80.6)	9888 (79.2)	
	≥1 time a week	606 (19.4)	2600 (20.8)	
Systolic blood pressure				0.001 *
	<120 mmHg	1013 (32.5)	4055 (32.5)	
	120–139 mmHg	1511 (48.4)	5693 (45.6)	
	≥140 mmHg	598 (19.2)	2740 (21.9)	
Diastolic blood pressure				0.030 *
	<80 mmHg	1536 (49.2)	5886 (47.1)	
	80–89 mmHg	1062 (34.0)	4274 (34.2)	
	≥90 mmHg	524 (16.8)	2328 (18.6)	
Fasting blood glucose				0.009 *
	<100 mg/dL	2182 (69.9)	8382 (67.1)	
	100–125 mg/dL	727 (23.3)	3124 (25.0)	
	≥126 mg/dL	213 (6.8)	982 (7.9)	
Total cholesterol				0.156
	<200 mg/dL	1628 (52.2)	6278 (50.3)	
	200–239 mg/dL	1043 (33.4)	4297 (34.4)	
	≥240 mg/dL	451 (14.5)	1913 (15.3)	
CCI score				<0.001 *
	0	1732 (55.5)	8917 (71.4)	
	1	744 (23.8)	1683 (13.5)	
	2	296 (9.5)	881 (7.1)	
	3	159 (5.1)	424 (3.4)	
	≥4	191 (6.1)	583 (4.7)	
Temporomandibular joint disorder		43 (1.4)	64 (0.5)	<0.001 *

Abbreviations: CCI—Charlson Comorbidity Index; * Chi-square test. Significance at *p* < 0.05† Obesity (BMI, body mass index, kg/m^2^) was categorized as <18.5 (underweight), ≥18.5 to <23 (normal), ≥23 to <25 (overweight), ≥25 to <30 (obese I), and ≥30 (obese II).

**Table 2:** Crude and adjusted hazard ratios (95% confidence interval) for temporomandibular joint disorder in the rheumatoid arthritis and control groups.

Characteristics		Hazard Ratios for Temporomandibular Joint Disorder			
		Crude †	*p*-Value	Adjusted †‡	*p*-Value
Total participants (*n* = 15,610)					
	Rheumatoid arthritis	2.70 (1.83–3.97)	<0.001 *	2.52 (1.70–3.74)	<0.001 *
	Control	1.00		1.00	
Age < 60 years old, men (*n* = 2045)					
	Rheumatoid arthritis	2.76 (0.78–9.80)	0.116	1.98 (0.47–8.35)	0.352
	Control	1.00		1.00	
Age < 60 years old, women (*n* = 6640)					
	Rheumatoid arthritis	2.13 (1.18–3.83)	0.012 *	1.96 (1.08–3.55)	0.027 *
	Control	1.00		1.00	
Age ≥ 60 years old, men (*n* = 2160)					
	Rheumatoid arthritis	4.77 (1.45–15.65)	0.010 *	5.97 (1.63–21.92)	0.007 *
	Control	1.00		1.00	
Age ≥ 60 years old, women (*n* = 4765)					
	Rheumatoid arthritis	3.06 (1.60–5.86)	0.001 *	2.98 (1.52–5.84)	0.001 *
	Control	1.00		1.00	

* Stratified Cox proportional hazard regression model, Significance at *p* < 0.05. † Models were stratified by age, sex, income, and region of residence. ‡ The model was adjusted for obesity, smoking, alcohol consumption, systolic blood pressure, diastolic blood pressure, fasting blood glucose, total cholesterol, and Charlson Comorbidity Index scores.
